# Real-Life Effects of Omalizumab on Chronic Rhinosinusitis with Nasal Polyposis

**DOI:** 10.3390/jpm14010003

**Published:** 2023-12-19

**Authors:** Nicola Lombardo, Giovanna Lucia Piazzetta, Nadia Lobello, Giuseppe Cicala, Maria Patafi, Anna Teresa Benincasa, Corrado Pelaia, Emanuela Chiarella, Girolamo Pelaia

**Affiliations:** 1 Otolaryngology Head and Neck Surgery, Department of Medical and Surgical Sciences, University “Magna Græcia”, 88100 Catanzaro, Italy; giovannapiazzetta@hotmail.it (G.L.P.); nadialobello@gmail.com (N.L.); giuseppe.cicala002@studenti.unicz.it (G.C.); anna.teresa84@hotmail.it (A.T.B.); 2 Department of Human Pathology, Division and School of Allergy and Clinical Immunology, University of Messina, 98100 Messina, Italy; mariapatafi@libero.it; 3 Department of Health Sciences, University “Magna Græcia”, 88100 Catanzaro, Italy; pelaia.corrado@gmail.com (C.P.); pelaia@unicz.it (G.P.); 4 Laboratory of Molecular Haematopoiesis and Stem Cell Biology, Department of Experimental and Clinical Medicine, University “Magna Græcia”, 88100 Catanzaro, Italy; emanuelachiarella@unicz.it

**Keywords:** chronic rhinosinusitis, nasal polyps, biologics, omalizumab, Sino-Nasal Outcome Test (SNOT-22), nasal polyp score (NPS), numeric rating scale (NRS), nasal congestion score (NCS)

## Abstract

Chronic rhinosinusitis with nasal polyposis (CRSwNP) is an inflammatory disease of the nasal and sinus mucosa. This inflammatory process is supported by a multitude of cytokines, including IL-4, IL-5, and IL-13 produced by Th2 cells, as well as by IgE produced by B lymphocytes in response to a stimulus. Omalizumab is an anti-IgE monoclonal antibody with well-recognized roles in allergic asthma and chronic spontaneous urticaria. The aim of this study was to evaluate the clinical efficacy of omalizumab in a cohort of 13 patients suffering from chronic rhinosinusitis with CRSwNP. The inclusion criteria considered were as follows: 18 years of age, with a diagnosis of chronic rhinosinusitis with severe nasal polyposis expressed by an NPS greater than or equal to 5 and/or a SNOT-22 greater than or equal to 50. In addition, in the enrolled patients, the classic treatment with corticosteroids had to have been suspended due to recurrence after surgery or lack of response. Our results highlighted that omalizumab treatment for 16 weeks improved the parameters analyzed: SNOT-22, NPS, NRS, and NCS. The clinical efficacy of omalizumab was further strengthened by a significant improvement in respiratory function as well as reductions in the nasal polyps’ size and in the associated symptoms.

## 1. Introduction

**Figure 1:**
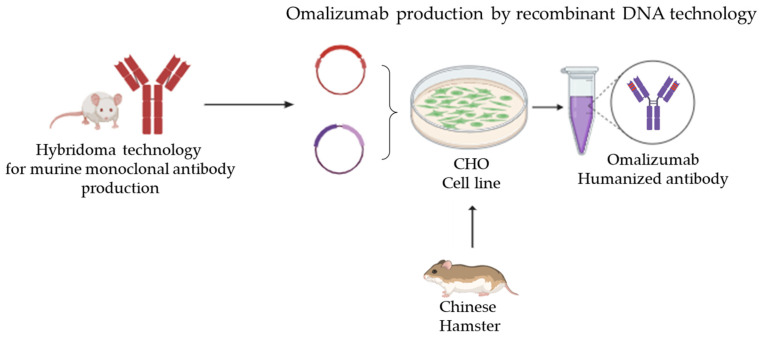
Schematic representation of omalizumab production by recombinant DNA technology: The heavy and light variable chains of the murine monoclonal antibody produced by hybridoma technology were cloned into an expression plasmid (red). Furthermore, the human light and heavy constant regions were also cloned into a second expression plasmid (purple). CHO cells obtained from a Chinese hamster were co-transfected by both plasmids, and the humanized monoclonal antibody produced in the medium was purified. The diagram was made using BioRender.

Chronic rhinosinusitis (CRS) is defined as chronic inflammation of the nose and paranasal sinuses [1]. According to the diagnostic criteria of the European Position Paper on Rhinosinusitis and Nasal Polyps 2020 (EPOS 2020), the diagnosis of chronic rhinosinusitis with nasal polyposis (CRSwNP) is confirmed by at least two of the following symptoms persisting for at least 12 weeks: nasal discharge, nasal congestion or obstruction, pain or pressure on the face, and decreased sense or loss of smell. Nasal polyps are inflammatory, benign lesions that protrude into the nasal cavities originating from the ethmoid sinus. They are formed by abundant edematous stroma rich in plasma proteins, especially albumin. The inflammatory environment and the epithelial-to-mesenchymal transition (EMT) play a fundamental role in the formation process of nasal polyps and, in particular, in nasal tissue remodeling. EMT is a complex mechanism in which epithelial cells loss their epithelial phenotype and acquire a mesenchymal one [2]. Interestingly, recent studies have evidenced the presence of a population of mesenchymal stem cells in nasal polyps [3,4]. In contrast, isolated nasal lesions occurring medial to the middle turbinate raise the suspicion of neoplasia. Specifically, CRSwNP is a disease of adulthood, with a mean age of onset of 42 years and a typical age of diagnosis between 40 and 60 years. However, evidence shows that women with CRSwNP exhibit more severe disease than men, and this is confirmed by significantly more pronounced radiographic evidence of sinus disease. The typical symptoms of CRSwNP that are usually reported by the patient are usually supported by objective evidence of sinus inflammation and nasal polyps through computed tomography (CT) and/or nasal endoscopy [5]. The presence of polyps in the nose significantly affects patients’ quality of life, compromising their work productivity and social life [6]. In an elegant study based on the 36-Item Multidimensional Health Questionnaire (SF-36), it was reported that the effect of CRS on bodily pain and social functioning was comparable or superior to that of other chronic diseases such as congestive heart failure, angina pectoris, chronic obstructive pulmonary disease, and chronic back pain [7]. CRS is classified into two main subgroups based on the results of endoscopy or computed tomography (CT): CRS without nasal polyps (CRSsNP) and CRS with nasal polyps (CRSwNP) [8]. Among all patients with CRS, only 25–30% have CRSwNP. CRSwNP is associated with significant morbidity and reduced quality of life [9,10]. Men are more likely than women to have CRSwNP [11,12]. CRSwNP is often associated with other important medical conditions that can influence the severity of the disease. In a retrospective study on a sample of over 400,000 patients in primary care, it was observed that patients diagnosed with CRSwNP had a significantly higher prevalence of pre-existing conditions such as acute rhinosinusitis, allergic rhinitis, chronic rhinitis, asthma, gastroesophageal reflux disease, and sleep apnea [13]. Most people with asthma (approximately 88%) have at least some radiographic evidence of inflammation of the nasal mucosa and sinuses [14]. A specific group of patients with CRSwNP and asthma may experience upper and/or lower respiratory symptoms after taking medications that inhibit the cyclooxygenase-1 enzyme. This condition is known as aspirin-exacerbated respiratory disease (AERD). It is estimated that approximately 10% of patients with nasal polyps and 9% of patients with CRS have AERD, although the true prevalence of this disease remains unknown [15]. Medical treatment options for patients with chronic nasosinusal polyposis (CRSwNP) remain limited. According to the most recent US guidelines, both intranasal corticosteroids and saline nasal irrigations are recommended as initial medical therapies for these patients [16]. Antibiotics may be useful in treating infections that aggravate CRSwNP, but significant evidence of clinical efficacy, such as reductions in the size and number of polyps, in large randomized trials is lacking [17]. In patients who experience worsening of symptoms or who cannot completely control their symptoms with standard therapies for CRSwNP, a short course of oral corticosteroids may be considered, but in patients who undergo frequent courses of oral corticosteroids there is an increased risk of numerous side effects, including osteoporosis or osteoporotic fracture, pneumonia, cataracts, and type 2 diabetes, compared to patients not taking courses of systemic corticosteroids [18]. Surgery is usually the next step when medical treatment is not sufficient to relieve symptoms in patients with CRSwNP [19]. The main procedure is functional endoscopic sinus surgery (FESS), which consists in the polyps’ deletion under direct visualization by optic fibers during nasal endoscopy. Unfortunately, recurrence is possible after surgery, and a recurrence rate of between 38% and 60% has been reported [20]. Although the pathophysiology of CRS is still under study, recent advances in understanding the inflammatory mechanisms involved have led to promising progress in the development of targeted biological therapies. Some biologics previously approved for the treatment of severe asthma have demonstrated therapeutic potential for the management of CRS, especially in patients with CRSwNP. The cytokines associated with type 2 inflammation, including IL-4, IL-5, and IL-13, along with the increased levels of IgE, have recently attracted considerable interest for their role in modulating eosinophilic airway inflammation in the condition of CRSwNP [21]. In the context of CRS treatment, the use of biologics requires careful evaluation of several factors, including disease severity, risk of polyp recurrence despite medical or surgical treatment, patient preferences and goals, safety, and cost–benefit ratio [22,23]. In particular, the presence of allergen-specific IgE on the surfaces of mast cells and basophils, once the antigen is bound, causes rapid degranulation of the cells. This involves the release of preformed inflammatory mediators and key cytokines such as IL-4 (which promotes the Th2 pathway and IgE production), IL-5 (which promotes eosinophil recruitment and survival), and IL-13 (which promotes the Th2 pathway and mucus production), contributing to the amplification and maintenance of existing inflammation [24]. Dendritic cells will present the allergen to naïve T lymphocytes, which, in an environment rich in IL-4 cytokines, will favor the development of Th2 cells. In turn, Th2 cells will secrete additional amounts of IL-4, IL-5, and IL-13, fueling an inflammatory cycle that further amplifies existing inflammation [25]. In the group of patients with type 2 CRSwNP, approximately 25–30% experience relapses after using oral corticosteroids or after conventional surgery, and they often require additional interventions throughout their lives [26]. Patients with CRSwNP who experience relapses after the use of systemic corticosteroids and/or after surgery have symptoms and a significant deterioration in quality of life (QOL), and they should be considered serious and uncontrolled patients, potentially candidates for treatment with new drugs with a high affinity for biological targets [27]. In recent times, several biological agents have been developed with the aim of inhibiting the Th2-type inflammatory response, which we find in most cases of CRSwNP. It has been hypothesized that in the future the patient’s endotype could be a predictive factor of their response to such biological therapies [28]. Among these, omalizumab is a humanized IgG1 monoclonal antibody that specifically binds human IgE. Although it still contains approximately 5% murine amino acids in the complementarity-determining regions (CDRs), it has been specifically modified to reduce the immune response against its murine component. Omalizumab binds to unbound IgE at the Cɛ3 domain [29]. Subsequently, through steric hindrance, omalizumab prevents IgE from interacting with its high-affinity receptor FcεRI on the cell surface, preventing subsequent activation and degranulation of mast cells and basophils. Free IgE is effectively neutralized, and inhibition of IgE-mediated signaling occurs rapidly [30]. Omalizumab received FDA approval in 2003 for the treatment of severe allergic asthma. Initially, it was prescribed to patients with severe asthma who demonstrated IgE sensitization to perennial allergens such as house dust mites or cats. However, after almost 17 years, awareness has emerged that IgE could play a functional role in CRSwNP, regardless of the atopic status of classical aeroallergenic allergens. The type 2 inflammatory pathways that are active in CRSwNP have been recognized to involve IgE-related inflammation [31]. Omalizumab binds to the Cε3 domain of IgE, a site that overlaps with binding sites for both FcεRI and FcεRII. Omalizumab can accelerate the dissociation of IgE from FcεRI on mast cells and basophils. FcεRI expression is closely correlated with serum IgE levels and unoccupied FcεRI sites on the cell surface. For this reason, treatment with omalizumab is able to determine a rapid reduction in the expression levels of FcεRI by mast cells, basophils, and antigen-presenting cells that normally express high levels of FcεRI on their cell surface [32]. The reduction in IgE and the parallel decrease in FcεRI expression limit the activation of effector cells and, thus, reduce IgE-mediated symptoms [33] (Figure 1). In this article, we report our experience regarding omalizumab treatment in a cohort of 17 patients with CRSwNP. The patients that we followed in this study were treated with omalizumab on the recommendation of the otolaryngologist, and not the pulmonologist, for asthma treatment. The data obtained are surprising, since after 16 weeks the omalizumab-treated patients had radical improvements in the symptoms associated with CRSwNP. In particular, the levels of SNOT-22, NPS, NRS, and NCS were significantly reduced compared to baseline. Although the patients enrolled in this study had mild or moderate asthma, they were still not candidates for treatment with biologics from a pneumological point of view. Instead, these patients satisfied the criteria for ENT indications.

## 2. Materials and Methods

### 2.1. Definition of Criteria for Enrollment of Patients to Be Treated with Omalizumab

The clinical efficacy of omalizumab was evaluated in a cohort of 13 patients (9 males and 4 females) aged between 41 and 79 years and affected by chronic rhinosinusitis with nasal polyposis. Twelve patients are continuing treatment, while one of them dropped out after the third administration due to a recent diagnosis of colorectal cancer. Eleven of the thirteen patients had a clinical diagnosis of asthma. 

The patients were treated with a 150 mg or 75 mg solution of omalizumab for subcutaneous injection with a prefilled pen every 2 or 4 weeks. The appropriate dose and frequency of administration of omalizumab for these clinical conditions can be determined by baseline IgE levels (IU/mL) and body weight (kg), according to the manufacturer’s instructions. Based on these determinations, 75 to 600 mg of omalizumab in 1 to 4 injections may be required for each administration. The enrolled patients had to be aged 18 years or older, have an established diagnosis of chronic rhinosinusitis with severe nasal polyposis, and have a disease severity expressed with a total endoscopic nasal polyposis score (NPS) ≥ 5 and/or SNOT-22 ≥ 50. In patients enrolled in this study, previous therapeutic treatments were discontinued due to lack of efficacy, as well as because systemic corticosteroid therapy did not respond or proved ineffective, leading to recurrence after surgery. Within the clinical sample examined, 11 patients underwent at least one FESS surgery, while 2 patients were excluded from surgery due to a personal refusal of surgical therapy and a high anesthetic risk, which made them unsuitable for the surgical procedure. These patients were directly referred to biological therapy. While evaluating the patients, several parameters were taken into consideration to understand the severity and impact of the disease. These parameters include the endoscopic nasal polyps score (NPS), which provides information on the extension and severity of nasal polyps. In addition, the enrolled patients were given the Sino-Nasal Outcome Test (SNOT-22) questionnaire developed at Washington University in St. Louis, Missouri, and the nasal congestion score (NCS), a subjective assessment of congestion symptoms, was calculated. Finally, the overall impact of the disease was assessed using a numerical scale called the numeric rating scale (NRS). This study was conducted in the period between February and June 2023. The study was carried out according to the standards of Good Clinical Practice and the principles of the Declaration of Helsinki. All enrolled patients signed a written informed consent form. The study was also conducted in agreement with a statement from the local ethical committee of the Calabria Region (Catanzaro, Italy; document 182—20 May 2021).

### 2.2. Nasal Polyps Score (NPS)

The nasal polyps score was performed using the PENTAX fiberscope. This approach allows us to obtain a score from 0 to 4; NPS values increase with the severity of the disease.

### 2.3. Sino-Nasal Outcome Test (SNOT-22)

The patients’ quality of life was assessed by the Sino-Nasal Outcome Test (SNOT-22) (Washington University, St. Louis, Missouri (MO), USA). This method consists of a test of 22 questions and allows the researcher to calculate a total score from 0 to 110. The quality of life of the CRSwNP patient is inversely proportional to the score obtained, so a high value is associated with a worse quality of life.

### 2.4. Nasal Congestion Score (NCS)

The nasal congestion score (NCS) is a parameter that is used to subjectively evaluate the severity of nasal congestion symptoms in a patient with CRSwNP. This score is obtained through the self-assessment of the patient theirself, who evaluates the severity of the congestion symptoms every morning, taking into consideration the previous 24 h. The NCS corresponds to the monthly average of the morning assessment carried out by the patient on the severity of their symptoms. The NCS is assessed using a 4-point scale according to the following criteria: 0 corresponds to the absence of symptoms (no obvious signs or symptoms); 1 corresponds to mild symptoms (clearly perceived presence of signs or symptoms, but with minimal awareness; easily tolerable); 2 indicates moderate symptoms (definite presence of signs or symptoms that are bothersome but still tolerable); 3 represents severe symptoms (presence of signs or symptoms that are difficult to tolerate, interfering with daily activities and/or sleep).

### 2.5. Numeric Rating Scale (NRS)

The NRS is a numerical rating scale used to evaluate the patient’s subjective perception of the disease. The NRS allows the researcher to obtain a score from 0 (“no problem”) to 10 (“worse perception of the disease”). There are three score ranges: 0 to 3 indicates mild symptoms, 4 to 7 indicates moderate symptoms, and 8 to 10 indicates severe symptoms [34].

### 2.6. Statistical Analysis

Different software tools were used to perform the statistical analysis, including Microsoft Excel, Prism 9.1.2 (GraphPad Software, San Diego, CA, USA), and Matplotlib. The results obtained were represented by the arithmetic mean followed by the standard deviation, indicated as mean ± SD. For all analyses conducted, a *p*-value < 0.05 was considered to be statistically significant.

## 3. Results

### 3.1. Gender and Age Distribution in Patients with CRSwNP

**Figure 2:**
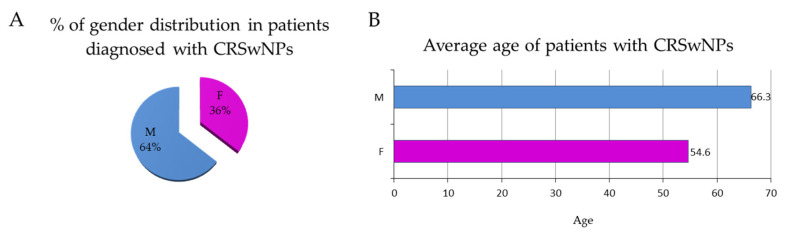
(**A**) The pie chart represents the percentage of gender distribution, and (**B**) the bar diagram indicates the average age of patients with CRSwNP.

The clinical efficacy of omalizumab was evaluated in a cohort of 13 patients, of whom 64% were males and 36% were females (Figure 2A). The mean age of the enrolled patients was 66.3 years old for men and 54.6 years old for women (Figure 2B).

### 3.2. Omalizumab Improved the SNOT-22, NPS, NRS, and NCS Parameters in Patients with CRSwNP 

**Figure 3:**
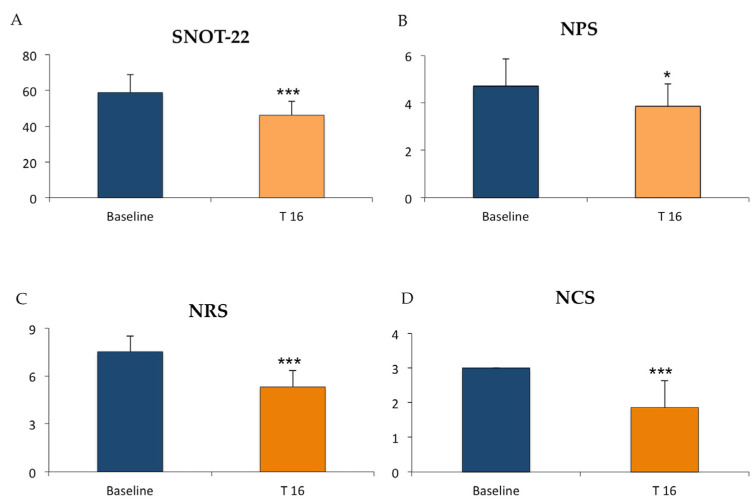
Diagrams indicate the mean values of (**A**) SNOT-22, (**B**) NPS, (**C**) NRS, and (**D**) NCS evaluated in 13 patients with CRSwNP treated with omalizumab for 16 weeks. The data were compared to the mean values at the baseline levels. Data are represented as means + SD from 13 patients (* *p* < 0.05, *** *p* < 0.0001).

Patients with an average IgE value at time 0 of 289.53 kU/L ± 224.3 were treated with omalizumab for 16 weeks. In these patients, SNOT-22, NPS, NRS, and NCS were evaluated. SNOT-22, the main predictor of chronic rhinosinusitis endoscopic sinus surgery, showed a reduction of about 1.34-fold in patients exposed to omalizumab for 16 weeks compared to baseline. Specifically, the mean SNOT-22 value at time 0 was 62.28 ± 15.43, while at 16 weeks it was 46.07 ± 7.93 (Figure 3A). NPS, indicating the severity of CRSwNP, was slightly improved; the average value was 4.71 ± 1.13 at time 0 and 3.84 ± 0.98 after 16 weeks of omalizumab treatment, with an overall reduction of 0.81-fold (Figure 3B). The evaluation of the numeric rating scale (NRS) instead resulted in an improvement of 1.42-fold, suggesting a lower perception of symptoms by the patient. In this case, the average value of the NRS was 7.57 ± 0.93 at the baseline vs. 5.3 ± 1.03 after the therapeutic approach (Figure 3C). Intriguingly, the nasal congestion score (NCS) parameter improved by 1.78-fold; the average value was 0: 3 ± 0 before omalizumab administration, while the average value was 1.77 ± 0.72 at 16 weeks. These data suggest that omalizumab had already reduced the severity of the patients’ nasal congestion after 16 weeks (Figure 3D).

### 3.3. Radar Graph Representation of the SNOT-22, NPS, NRS, and NCS Parameters in Patients with CRSwNP

**Figure 4:**
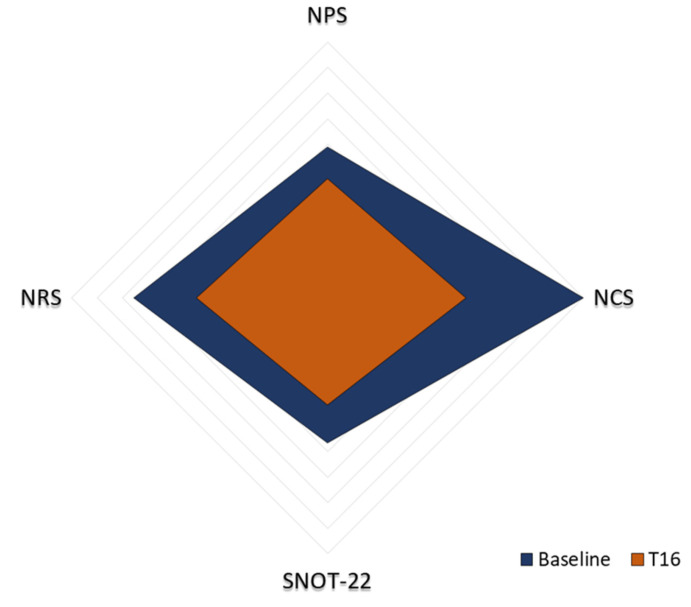
Radar graph representation. The area of the dark blue quadrilateral indicates the baseline values of SNOT-22, NPS, NRS, and NCS. The area of the orange parallelogram shows data obtained after 16 weeks of omalizumab treatment. The graph was made using PowerPoint 2009.

The parameters analyzed in this study were mapped on a single axis. A radar graph was realized to provide an immediate visual representation of the trend of SNOT-22, NPS, NRS, and NCS over time (Figure 4). The key to reading this graph consists in analyzing the area of the quadrilateral formed by the axes. Specifically, the parallelogram in light orange indicates the baseline of the considered parameters; conversely, the dark blue parallelogram refers to data obtained after 16 weeks of omalizumab treatment. There is an evident reduction in the area of the quadrilateral corresponding to the instant T/16 compared to that at the instant T/0. This result directly reflects the decrease in the four variables analyzed in this study, providing an immediate indication of the extent of the variation observed.

### 3.4. Analysis of the SNOT-22, NPS, NRS, and NCS Parameters’ Variations

**Figure 5:**
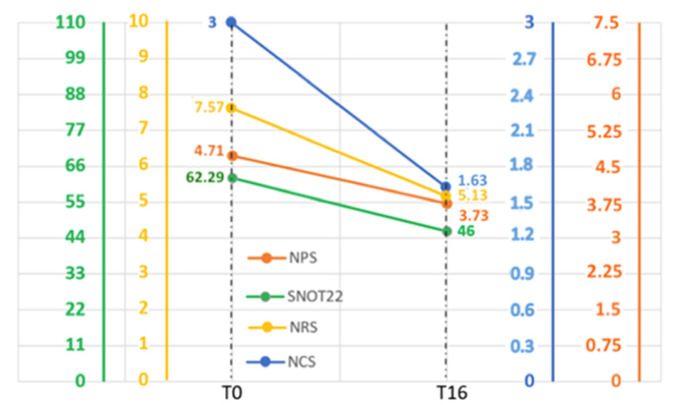
The graph shows the trends of the 4 variables over time. The upper limit indicates the worst scenario at baseline (SNOT-22: 62.29, NPS: 4.71, NRS: 7.57, and NCS: 3), while the lower limit (SNOT-22: 46, NPS: 3.73, NRS: 5.13, and NCS: 1.63) indicates the best scenario after 16 weeks of omalizumab treatment. The graph was made using PowerPoint 2009.

The graph in Figure 5 shows the trend of the four variables over time. For each parameter, the corresponding range of values is reported: the upper limit indicates the worst scenario at baseline (SNOT-22: 62.29, NPS: 4.71, NRS: 7.57, and NCS: 3), while the lower limit (SNOT-22: 46, NPS: 3.73, NRS: 5.13, and NCS: 1.63) indicates the best scenario after 16 weeks of omalizumab treatment. Furthermore, by comparing the SNOT-22, NPS, NRS, and NCS values at T0 and T16, we obtained the percentage change compared to the maximum value by using the following formula: (X_T0 − X_(T16))/X_max, where X represents the value of the parameter considered (Figure 5). In particular, in our study group, the percentage variations were as follows—SNOT-22: −14%, NPS: −12%, NRS: −24%, NCS: −45%.

## 4. Discussion

Omalizumab is a humanized IgG1 monoclonal antibody that specifically binds human IgE. Although it still contains approximately 5% murine amino acids in the complementarity-determining regions (CDRs), it has been specifically modified to reduce the immune response against its murine component. Omalizumab binds to unbound IgE at the Cɛ3 domain [35]. Subsequently, through steric hindrance, omalizumab prevents IgE from interacting with its high-affinity receptor FcɛRI on the cell surface, preventing subsequent activation and degranulation of mast cells and basophils. Free IgE is effectively neutralized, and inhibition of IgE-mediated signaling occurs rapidly [36]. In 2003, omalizumab received FDA approval for the treatment of severe allergic asthma. Initially, it was prescribed to patients with severe asthma who demonstrated IgE sensitization to allergens such as house dust mites or cats. However, after almost 17 years, awareness has emerged that IgE could play a functional role in nasal sinus polyposis (CRSwNP), regardless of the atopic status of classical aeroallergens [37]. The type 2 inflammatory pathways that are active in CRSwNP have been recognized to involve IgE-related inflammation. 

Some placebo-controlled randomized trials (NCT03478930, NCT01066104, NCT03280550) have reported the efficacy of omalizumab for the treatment of chronic rhinosinusitis with nasal polyposis [38,39,40]. These investigations highlighted a reduction in the volume of polypoid mucosal tissue, while the total symptom score was significantly improved. In addition, omalizumab treatment was able to prevent nasal tissue eosinophilia. In particular, in two randomized phase 3 trials, POLYP 1 and POLYP 2, Gevaert et al., analyzed omalizumab’s safety and efficacy in CRSwNP. In these studies, CRSwNP patients who did not respond to corticosteroid therapy were randomized to omalizumab or placebo combined with intranasal mometasone for 24 weeks respectively. Omalizumab treatment resulted in a significant reduction in specific clinical parameter values, including NPS, NCS, and SNOT-22. Specifically, the mean changes compared to baseline at week 24 for omalizumab treatment versus placebo were as follows: NPS, −1.08 vs. 0.06 (*p* < 0.0001) and −0.90 vs. −0.31 (*p* < 0.0140); nasal congestion score, −0.89 vs. −0.35 (*p* < 0.0004) and −0.70 vs. −0.20 (*p* < 0.0017); SNOT-22 score, −24.7 vs. −8.6 (*p* < 0.0001) and −21.6 vs. −6.6 (*p* < 0.0001) [41]. In addition, these data were confirmed by Damask et al., in subgroups of patients pooled from the POLYP 1 and POLYP 2 studies. Treatment with omalizumab showed no adverse events in patients with CRSwNP, thus showing a good safety profile similar to those reported in severe asthma studies with omalizumab over ten years [42]. Similar to the results published in the POLYP 1 and POLYP 2 clinical trials, our data demonstrate the effectiveness of omalizumab treatment to improve the CRSwNP patients’ clinical symptoms and quality of life after 16 weeks. In fact, all of the parameters analyzed (NPS, SNOT-22, NCS, and NRS) improved after the treatment. In particular, the nasal polyp score (NPS) decreased, indicating a reduction in the size of the polyps. With considerable patient satisfaction, our data showed an improvement in olfactory function, and this aspect was highlighted by the reduction in SNOT-22 scores. The improvement in the quality of life of our patients was further confirmed by the decreases in the values of the NRS and NCS for the subjective perception of the disease. Our data are supported by the work of Maza-Solano et al., who demonstrated the clinical efficacy of omalizumab when combined with ESS treatment. In this case, the analyzed patients showed a notable improvement in symptoms and quality of life as early as the sixteenth week. These clinical benefits persisted over 2 years [43]. The efficacy of omalizumab was also demonstrated in a real-life study conducted by Tuğba Songül Tat. Particularly, treatment with this IgE-targeted monoclonal antibody improved symptoms in 17 patients with recalcitrant CRSwNP with or without asthma. Specifically, the SNOT-22 score, nose score, VAS for postnasal drip, rhinorrhea, stuffy nose, odor, and sneezing were significantly reduced from baseline. Thanks to the treatment, the patients experienced a significant reduction in the volume of polyps, helping to improve the patency of the nasal passages. Interestingly, the efficacy of omalizumab does not manifest as quickly as that of dupilumab; however, the effect of omalizumab is maintained over time [44,45,46]. Confirming the current evidence from the literature, the safety data were overall consistent with the known safety profile of omalizumab; in fact, the drug was well tolerated in our cohort of patients, with the complete absence of side effects linked to the treatment. Further larger-scale studies and long-term reevaluation are needed to confirm these findings; however, the current data clearly indicate the efficacy and safety of omalizumab as a promising therapy for CRSwNP. Analyzing the criteria variations in the study cohort considered, we can strongly affirm that omalizumab has already demonstrated its effectiveness in reducing all of the considered variables after 16 weeks of evaluation. The efficacy of omalizumab in allergic asthma, and now in CRSwNP, highlights the central role of IgE in regulating the recruitment and function of cells that mediate type 2 inflammation. Omalizumab studies confirm that IgE-mediated sensitization to aeroallergens is irrelevant to the clinical outcomes of isolated or asthma-associated CRSwNP, supporting a central role for IgE localized to the airway mucosa. In fact, by occupying the binding sites for both FcεRI and FcεRII, omalizumab is able to accelerate the dissociation of IgE from FcεRI on mast cells and basophils. FcεRI expression is closely correlated with serum IgE levels and unoccupied FcεRI sites on the cell surface. For this reason, treatment with omalizumab is able to cause a rapid reduction in the expression levels of FcεRI by mast cells, basophils, and antigen-presenting, cells which normally express high levels of FcεRI on their cell surface. Reductions in IgE and parallel FcεRI expression limit the activation of effector cells and, thus, reduce IgE-mediated symptoms in CRSwNP.

## 5. Conclusions

**Figure 6:**
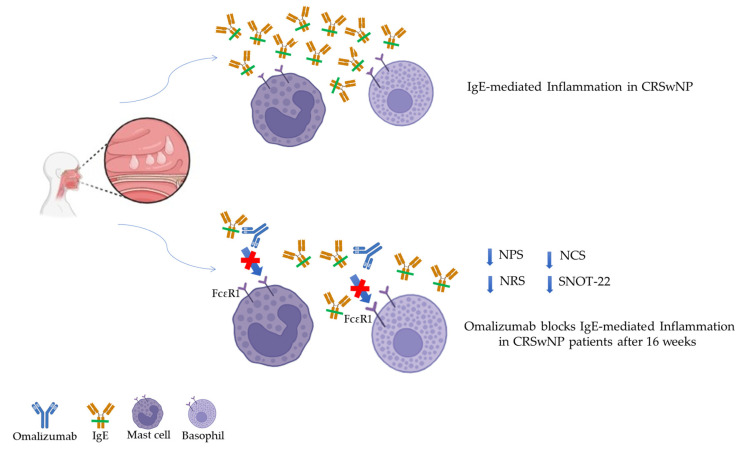
Schematic representation of the effects of omalizumab on 13 patients with CRSwNP. CRSwNP is an inflammatory disease in which IgE-mediated inflammation plays a central role. Omalizumab, a humanized monoclonal antibody, occupies the binding sites for both FcεRI and FcεRII. Omalizumab is able to accelerate the dissociation of IgE from FcεRI on mast cells and basophils. Treatment with omalizumab for 16 weeks led to a marked reduction in the Sino-Nasal Outcome Test-22 (SNOT-22), nasal polyp score (NPS), numeric rating scale (NRS), and nasal congestion score (NCS). The diagram was made using BioRender. Red Cross symbol indicates the blocking of FcɛRI on IL-4 and IL-13 receptors by omalizimab.

Chronic rhinosinusitis with nasal polyps (CRSwNP) is a chronic and debilitating condition characterized by a high symptom burden, with a significant impact on quality of life (QoL), allergic comorbidities such as asthma, and respiratory disease exacerbated by NSAIDs (aspirin-exacerbated respiratory disease). In a context in which classic therapies are often ineffective or insufficient for controlling CRSwNP, the advent of new biologics represents a further tool to control the disease and potentially cause its remission. In a cohort of 13 patients with CRSwNP, we investigated omalizumab’s efficacy by analyzing four key variables (NPS, SNOT-22, NCS, and NRS) (Figure 6). The results obtained were very encouraging; they evidenced a significant improvement in all four variables studied. In particular, patients treated with omalizumab reported a notable reduction in symptoms associated with chronic rhinosinusitis, including nasal congestion, itching, rhinorrhea, and sensations of pressure or pain in the paranasal sinus region, reducing the need for systemic corticosteroid therapy. Furthermore, omalizumab has demonstrated a positive effect on reducing the size of nasal polyps.

## Data Availability

Data are contained within the article.

## Abbreviations

CRSwNP	CRS with nasal polyps
CRSsNP	CRS without nasal polyps
EPOS	European Position Paper on Rhinosinusitis and Nasal Polyps
ENT	Ear, nose, and throat
NPS	Nasal polyp score
SNOT-22	Sino-Nasal Outcome Test
NRS	Numeric Rating Scale
NCS	Nasal congestion score
CRS	Chronic rhinosinusitis
EMT	Epithelial-to-mesenchymal transition
CT	Computed tomography
AERD	Aspirin-exacerbated respiratory disease
FESS	Functional endoscopic sinus surgery
QoL	Quality of life
CDRs	Complementarity-determining regions
NSAIDs	Nonsteroidal anti-inflammatory drugs
VAS	Visual analogue scale
ESS	Endoscopic sinus surgery
